# 

**Published:** 1876-05

**Authors:** 


### Meetings of Medical Societies.

The meeting of the American Medical Association is to be held in Philadelphia, Tuesday, June 6th, 1876. Dr. J. Marion Sims, President.

The annual meeting of the New York State Medical Society will be held in Albany, Tuesday, June 13th, 1876. Dr. Thos. F. Rochester, President. It is hoped there will be a full attendance. In this connection we give the following list of delegates from the New York State Medical Society to the International Medical Congress to be held at Philadelphia, Sept. 4th–9th, 1876 :

| | |
|---|---|
| Frank H. Hamilton | New York. |
| B. Fordyce Barker | New York. |
| Edward H. Parker | Poughkeepsie. |
| Frederick Hyde | Cortland Village. |
| Henry W. Dean | Rochester. |
| John P. Gray | Utica. |
| L. I. Teft | Syracuse. |
| J. V. P. Quackenbush | Albany. |
| James P. White | Buffalo. |
| George Burr | Binghamton. |
| S. O. Vanderpoel | Quarantine. |
| G. A. Dayton | Mexico, Oswego Co. |
| Wm. C. Wey | Elmira. |
| C. R. Agnew | New York. |
| B. F. Sherman | Ogdensburg. |
| E. M. Moore | Rochester. |
| J. C. Hutchinson | Brooklyn. |
| Francis Burdick | Johnstown, Fulton Co. |
| Geo. J. Fisher | Sing Sing. |
| Harvey Jewett | Canandaigua. |
| Ellsworth Eliot | New York. |
| Julius F. Miner | Buffalo. |
| W. H. Bailey | Albany. |
| E. R. Hun | Albany. |
| C. H. Porter | Albany. |
| J. Foster Jenkins | Yonkers. |
| A. M. Vedder | Schenectady. |
| H. D. Didama | Syracuse. |
| E. R. Squibb | Brooklyn. |
| Alexander Hutchins | Brooklyn. |
| H. D. Vosburg | Lyons. |
| Austin Flint, Jr. | New York. |
| John D. Button | Auburn. |
| Thomas M. Flandreau | Rome. |

The Semi-Annual Meeting of the Medical Society of the County of Erie, will be held at the Buffalo Medical College, Tuesday, June 13th, 1876. A full attendance is desirable.

### Books and Pamphlets Received.

Lecture on Orthopædic Surgery and Diseases of Joints. Delivered at Bellevue Hospital Medical College, Session of 1874-75. By Lewis A. Sayre, M. D., Illustrated by 274 wood engravings. New York: D. Appleton & Co. 1876. Buffalo: Herger & Ulbrich.

Extracts from the Ninth Annual Report of the State Board of Charities of New York State, relating to Hospitals for the Sick and Insane. By M. B. Anderson and J. C. Devereux. To which is appended a Report relating to the Management of the Insane in Great Britain. By H. B. Wilbur, M. D.

Remarks on Urethral Stricture, before the British Medical Association, Aug. 5th, 1875. By F. N. Otis, M. D. of N. Y. Reprint from *British Medical Journal*, Feb. 26th, 1876.

### Contents of May Number.

### ORIGINAL.

THE BUFFALO

### Medical and Surgical Journal

PUBLISHED MONTHLY,

Containing Original Articles, Reports of Medical Societies and Hospitals, Editorial, Reviews, Correspondence, Army News, etc., etc; including the usual variety of Medical Periodical Publications. Specimen copies sent on application.

Address Buffalo Medical & Surgical Journal, Buffalo, N. Y.

TERMS OF SUBSCRIPTION, $3.00 PER YEAR, IN ADVANCE.

